# Recurrence patterns and management of locally recurrent rectal cancer: a retrospective cohort study

**DOI:** 10.1007/s00423-025-03692-x

**Published:** 2025-04-02

**Authors:** P. Hakenberg, G. Kalev, S. Seyfried, C. Reißfelder, J. Hardt

**Affiliations:** 1 Department of Surgery, Medical Faculty Mannheim, University Medical Center Mannheim, Heidelberg University, Theodor-Kutzer-Ufer 1-3, D-68167 Mannheim, Germany; 2 Medical Faculty Mannheim, DKFZ-Hector Cancer Institute, Heidelberg University, Mannheim, Germany

**Keywords:** Recurrent rectal cancer, Colorectal cancer, Abdominoperineal resection, Complications, Survival

## Abstract

**Purpose:**

Treatment of locally recurrent rectal cancer (LRRC) is still challenging because of inhomogeneous patient cohorts regarding previous treatments as well as different recurrence patterns and locations. The aim of this study was to investigate the treatments and surgical approaches tailored to them.

**Methods:**

We included all patients who were treated for LRRC without distant metastasis at the University Medical Center Mannheim, Germany, between 2010 and 2021. We collected data from our electronic clinical data management system regarding the initial diagnosis and treatment, as well as the locations and treatment of the recurrent tumor.

**Results:**

We identified a total of 666 patients who were curatively treated for rectal cancer of whom 36 patients (5.4%) developed LRRC without distant recurrence. Most patients (26/36) had a tailored therapy regimen that included surgery with or without perioperative radiation and/or chemotherapy. The most common site of local relapse was around the former colorectal anastomosis (15/36, 41.7%). The operative procedures ranged from anterior resection to multi-organ resection and exenteration. A complete resection (R0) could be achieved in twelve patients (12/22. 54.5%). The 3- and 5-year overall survival rates were 79% and 72%, respectively.

**Conclusion:**

Most local recurrences occur at the anastomotic site and are mostly eligible for curative surgical therapy with good long-term survival.

## Introduction

Rectal cancer is one of the most common cancers in the world and, in 2020, 12.7% of all new cancer diagnoses were colorectal cancer. Due to far-reaching advances in surgical procedures, and radio- and chemotherapy as well as improved screening and diagnostics, survival and recurrence rates could be improved over the last decades [1]. Recurrence rates ranged from approximately 5% to up to 30% [2, 3], but could be reduced due to above mentioned advances to 4–10% [3–6]. The treatment of locally recurrent rectal cancer (LRRC) remains challenging because of inhomogeneous patient cohorts regarding previous treatments as well as different patterns and locations of recurrence. Therefore, LRRC is an ongoing topic of concern because of the increasing incidence of colorectal cancer especially in patients of young age with a relatively long life expectancy and thus a higher chance of local or disseminated cancer recurrence as stated in the recently published colorectal cancer statistics in the US [7] and all over the world [8].

There is no clear evidence on how to treat LRRC properly, and most studies are retrospective and have a small sample size. To our knowledge, up to now there is no prospective study regarding treatment of LRRC and only one national guideline [9].

The aim of this study was to investigate LRRC patterns as well as multimodal treatments and surgical approaches tailored to them.

## Methods

In this retrospective study, all patients treated for LRRC without the presence of distant metastases at the University Medical Center Mannheim, Germany, between 2010 and 2021 were included. This encompassed all patients with LRRC irrespective of whether their primary surgeries were conducted externally or within our institution. To identify all patients meeting the inclusion criteria, we searched our clinical information system using the ICD (International Statistical Classification of Diseases and Related Health Problems) Code C20, due to the absence of a specific code for LRRC and manually excluded all primary non-curative and non-operative cases. The remaining cases were evaluated for LRRC, and only those without distant metastases were incorporated into the analysis. Primary non-curative cases were defined as those with irresectable distant metastases, which therefore did not receive surgical resection of the primarius but systemic therapy. By definition, these patients could not suffer a relapse without prior radical surgical resection of the primary tumor. Individuals who exhibited concurrent development of distant metastases with local recurrence were excluded as well to ensure the examination of a cohort with maximal homogeneity. Recurrence was assessed via magnetic resonance imaging (MRI) or CT scans, or by endoscopic examination where applicable. Data collection encompassed baseline and oncological characteristics, including prior systemic and radiation therapies, pathological results from the point of initial surgery, the first confirmed evidence of recurrent cancer, tumor localization in CT or MRI scans, and information regarding recurrent cancer treatment and survival data. The localization of recurrence was classified utilizing the Memorial Sloan-Kettering Classification System [10].

This study obtained approval from the local ethics committee (ID: 2022-812-AF 11). Due to the retrospective design of the study, patient consent was deemed unnecessary.

Descriptive statistics and survival analysis were performed using Microsoft Excel (Version 2024 Microsoft Corporation, Redmond, WA, USA).

## Results

Through screening of our clinical data management system for C20, we identified a total of 699 patients diagnosed with rectal cancer between January 2010 and June 2021 of which 666 were curatively treated for rectal cancer. Among these, 37 had LRRC, due to simultaneous distant tumor recurrence, we excluded one of the 37 patients. The remaining 36 patients (36/666, 5.4%) with isolated LRRC were included in our analysis.

The median age at the time of the first curative surgery was 62.5 years (IQR (interquartile range): 53–72). A complete resection, classified as R0, was accomplished in 32 patients (32/36, 88.9%). No patient had a macroscopic incomplete resection (R2). However, two patients, accounting for 5.6% (2/36, 5.6%) had a microscopically incomplete resection (R1). The R1 status was observed in patients whose T status was classified as 4, and in one of these individuals, a local tumor perforation had occurred. Both patients underwent a low anterior resection with a laparoscopic approach. Additionally, the patient who experienced tumor perforation required concurrent hysterectomy and ureteral reconstruction due to tumor infiltration.

Twenty-four patients (24/36, 66.7%) received a (low) anterior resection, with a majority undergoing laparoscopic surgery (21/36, 58.3%). A total of 4 patients (4/36, 11.1%) were eligible for a local procedure such as endoscopic submucosal dissections or transanal surgery. Abdominoperineal resection was necessary for eight patients due to the extremely low position and the advanced stage of their tumors.

Postoperative complications occurred in eleven patients (11/36, 30.6%). A singular case of anastomotic leackage necessitated endoscopic intervention via endoluminal vacuum therapy. Wound healing deficiencies were identified in seven patients (7/36, 19.4%) of whom five (5/36, 13.9%) experienced secondary wound healing in the sacral region following abdominoperineal resection, which was the most common complication after this type of surgery. Two patients (2/36, 5.6%) developed postoperative ileus and required temporary placement of a nasogastric tube. Non-surgical complications including pneumonia and cardiac diseases, manifested in five patients (5/36, 13.9%). Table 1 provides a summary of all characteristics pertinent to patients, tumors and treatments regarding the primary rectal cancer diagnosis.

**Table 1:** Patient, procedure and tumor characteristics at the time of the primary tumor resection

Characteristic	Median or absolute number	IQR^a^ or percentage
**Age (years)**	62.5	53–72
**Gender Distribution m: f**	22:14	61.1%:38.9%
**Neoadjuvant therapy**		
Radio-Chemotherapy	15	41.7%
Radiotherapy only	3	8.3%
No neoadjuvant therapy	18	50.0%
**Initial Surgical Procedures**		
AR^b^	5	13.9%
LAR^c^	19	52.8%
APR^d^	8	22.2%
Local Procedures	4	11.1%
Other	0	0.0%
**Approaches**		
Laparoscopic	21	58.3%
Open	7	19.4%
Robotic	0	0.0%
Not Applicable (local procedure)	4	11.1%
unknown	4	11.1%
**UICC Staging**		
I	6	16.7%
IIA	10	27.8%
IIB	3	8.3%
IIIA	2	5.6%
IIIB	7	19.4%
IIIC	3	8.3%
IV	3	8.3%
unknown	2	5.6%
**T Staging**		
T1	2	5.6%
T2	8	22.2%
T3	18	50.0%
T4	6	16.7%
unknown	2	5.6%
**N Status**		
N0	20	55.6%
N1	7	19.4%
N2	4	11.1%
Not applicable	4	11.1%
unknown	1	2.8%
**Grading**		
G1	2	5.6%
G2	18	50.0%
G3	4	11.1%
unknown	12	33.3%
**R Status**		
R0	32	88.9%
R1	2	5.6%
R2	0	0.0%
unknown	2	5.6%
**Regression grade (Dworak)**		
Grade 1	7	38.9%
Grade 2	6	33.3%
Grade 3	2	11.1%
Grade 4	0	0.0%
unknown	3	16.7%
Not applicable	18	50.0%

^a^ interquartile range, ^b^ abdominoperineal resection, ^c^ anterior resection, ^d^ low anterior resection

### Local recurrence patterns and outcomes after surgery for LRRC

The median time to recurrence of rectal cancer in our cohort was 20.5 months (IQR: 16.75–30) with a range of 2 months to 84 months. Table 2 presents tumor and treatment characteristics at the time of tumor recurrence.

**Table 2:** Tumor and treatment characteristics at the time of tumor recurrence

Characteristic	Median or absolute number	IQR^a^ or percentage
**Time till Recurrence** (months)	20.5	16.75-30
**Grading**		
G1	0	0.0%
G2	9	25.0%
G3	1	2.8%
unknown	26	72.2%
**Multimodal Therapy**		
Surgery only	7	19.4%
Surgery + Chemo-/Radiotherapy	18	50.0%
Chemo-/Radiotherapy only	9	25.0%
Best Supportive Care	0	0.0%
Unknown	2	5.6%
**Operative Procedure**		
APR^b^	6	23.1%
APR^b^, Sacrectomy	1	3.8%
APR,^b^ Adjacent Organ Resection	2	7.7%
LAR^c^	4	15.4%
LAR^c^, Adjacent Organ Resection	0	0.0%
Pelvic Exenteration	3	11.5%
Pelvic Exenteration, Sacrectomy	2	7.7%
Palliative Surgery	4	15.4%
Other	4	15.4%
**Intraoperative Radiotherapy**:		
Yes	6	23.1%
No	20	76.9%
Not Applicable	10	27.8%
**R Status**:		
R0	12	54.5%
R1	9	40.9%
R2	0	0.0%
unknown	1	4.5%

^a^ interquartile range, ^b^ abdominoperineal resection, ^c^ low anterior resection

In total, 26 patients (26/36, 72.2%) were subjected to a therapy regimen for their local recurrence that included surgery. Of these, seven patients (7/36, 19.4%) were treated with surgery only, while 19 patients (19/36, 52.8%) received a combination of radiotherapy, chemotherapy and surgery. Eight patients (8/36, 22.2%) were treated with either radiation therapy and/or chemotherapy exclusively. No patient received best supportive care. For two patients (2/36, 5.6%), the further course of the disease is indeterminate, as they declined any further diagnostics and treatment.

The operative group covers a wide range of procedures: nine abdominoperineal resections (9/26, 34.6%), one with sacrectomy (1/26, 3.8%), and two involving additional organ resection (2/26, 7.7%), alongside four low anterior resections (4/26, 15.4%). All of these patients had previously undergone either anterior or low anterior resection during their initial surgical treatment. Complete exenteration was necessary for five patients (5/26, 19.2%).

Most recurrences occurred around the anastomosis, with a total of 15 patients (15/36, 41.7%). Of these 15 patients, five patients (5/15, 66.7%) exhibited tumor mass within the former tumor region and anteriorly with infiltration into adjacent structures such as the vagina, bladder, or prostate, while one patient presented with tumor recurrence in the former tumor region accompanied by lateral extension. Five patients had only anterior recurrence, and four presented only posterior tumors with sacral infiltration. Again, for two patients (2/36, 5.6%), the specific anatomical site of tumor recurrence remains undefined, due to their refusal of further diagnostic evaluation. Notably, only one of the cohort of 36 patients with LRRC experienced anastomotic leakage after the primary surgery. It is noteworthy that the local recurrence in this particular case did not manifest in the area of the prior anastomosis, but rather appeared posteriorly and laterally, potentially correlating with the anatomical region of the previous leakage cavity.

Among the subset of patients who underwent surgical resection of their LRRC, an R0 resection was achieved in twelve individuals (12/22, 54.5%).

Nine patients (9/36, 25.0%) had tumor recurrence only in the region of the anastomosis or where the former tumor was located without involvement of adjacent organs or presence tumor mass elsewhere beyond the anastomotic site. Among these nine patients, two underwent an anterior resection as the initial surgical intervention, two had a local resection, feasible through transanal access, and the remaining five patients underwent a low anterior resection as the initial procedure. Eight out of these nine patients qualified for a second surgery to address their recurrent cancer. Both patients who previously underwent local transanal tumor resection subsequently received an abdominoperineal resection. Three patients had a low anterior resection again, one patient with recurrent anastomotic tumor mass underwent palliative stoma placement due to colon ileus and another patient had a low anterior resection with additional ileocecal resection. Table 3 illustrates the recurrence sites alongside their corresponding multimodal treatment strategies.

**Table 3:** Locations of tumor recurrence and corresponding multimodal treatments

			Previous treatment (*n*)		Therapy for LRRC^a^ (*n*)			
Localisation (Memorial Sloan Kettering Classification)	n	%	Neoadjuvant	Adjuvant	RT^b^/CT^c^	RT^b^/CT^c^/Surgery	Surgery	IORT^d^
Axial	9	25.0%	1	2	1	6	2	0
Anterior	5	13.9%	4	3	1	2	2	1
Posterior	4	11.1%	2 (1x only RT)	3	0	3	1	2
Lateral	5	13.9%	5 (1x only RT)	2	2	2	0	1
Axial + anterior	5	13.9%	2	3	0	4	1	2
Axial + lateral	1	2.8%	0	1	0	0	1	0
Posterior + lateral	3	8.3%	2	1	2	1	0	0
Anterior + posterior	1	2.8%	1	0	0	1	0	0
Unknown	3	8.3%						

^a^ locally recurrent rectal cancer, ^b^ radiotherapy, ^c^ chemotherapy; ^d^ intraoperative radiotherapy

Intraoperative radiation therapy was administered to six patients (6/26, 23.1%), all of whom presented with a complex tumor recurrence pattern encompassing lateral positions and infiltration into adjacent organs. Two patients required pelvic exenteration, one patient needed exenteration with sacrectomy, another patient underwent sacrectomy alone, and two patients underwent abdominoperineal resection. Table 4 presents the surgical approach depending on the site of recurrence.

**Table 4:** Locations of tumor recurrence and corresponding surgical procedures

Localisation	Procedures (*n*)								
	APR^a^	APR^a^, sacretomy	APR^a^, adjacent organres.	LAR^b^	LAR^b^ + adjacent organres.	Pelvic exenteration	Pelvic exenteration + sacrectomy	Palliative surgery	Other
Axial	2	0	0	4	0	0	0	1	1
Anterior	0	0	0	0	0	2	1	1	0
Posterior	1	0	0	0	0	0	1	1	1
Lateral	1	0	0	0	0	0	0	0	1
Axial + anterior	1	0	2	0	0	1	0	1	0
Axial + lateral	0	0	0	0	0	0	0	0	1
Posterior + lateral	1	0	0	0	0	0	0	0	0
Anterior + posterior	0	1	0	0	0	0	0	0	0

^a^ abdominoperineal resection, ^b^ low anterior resection

The postoperative complication rate following LRRC resection was 57.7% (15 patients suffered at least one postoperative complication), and complications were various. One of the three patients (1/3, 33.3%) who underwent low anterior resection without restoration of bowel continuity had leakage of the rectal stump necessitating intervention via endoluminal vacuum therapy. Among fourteen patients who underwent abdominoperineal resection or pelvic exenteration, seven patients (7/14, 50.0%) exhibited perineal wound healing disorder. Of these fourteen patients, nine (9/14, 64.3%) received an omentoplasty, one patient each received a VRAM (vertical rectus abdominis myocutaneous) flap or gluteal flap (each 7.1%), while in three patients (3/14, 21.4%) no omentoplasty was used and primary closure of the perineal wound was performed. Five perineal wound infections occurred after omentoplasty, one following a gluteal flap and one post-primary closure of the perineal wound. Postoperative ileus occurred in two patients (2/26; 7.7%) and non-surgical complications including respiratory disorders or renal failure were found in one patient each (3.8% each).

**Fig. 1:**
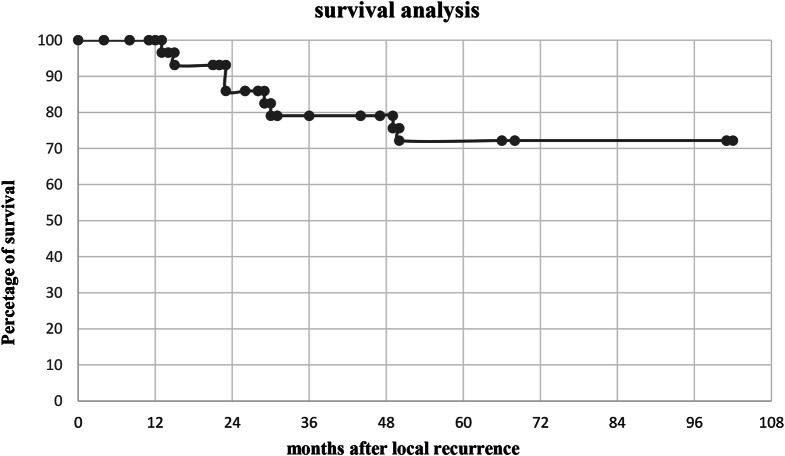
Survival data of our cohort

For survival analysis, six patients were lost to follow up. Thus, the remaining 30 patients were analyzed with a median follow up of 23 months (IQR: 12–30 months). The overall survival rates at 3 and 5 years were 79% and 72%, respectively. Figure 1 illustrates the cohort’s survival over time through a Kaplan-Meier curve.

## Discussion

This retrospective analysis of an almost 12-year period at a colorectal surgery center of excellence within a German university hospital revealed a local recurrence rate following curative treatment for rectal cancer of 5.4% (36/666). This is consistent with the recent literature which reports a recurrence rate ranging from 4 to 11% [2, 9, 11, 12]. The foremost and, thus far, most significant surgical milestone in the development of rectal cancer surgery was the introduction of “total mesorectal excision” by Heald in the second half of the twentieth century [13]. Heald successfully attained a local recurrence rate of less than 4%, which was previously deemed unachievable. Most recurrent tumors have either simultaneous local and distant recurrence or solely distant recurrence [2, 11, 12, 14]. Jung et al. [2] who executed a retrospective analysis of risk factors for local recurrence over a span of 15 years, divided their cohort into three groups based on the time of the primary operation. The analysis revealed a decline in the recurrence rate over time from up to 13% (2002–2006) down to only 5% (2012–2017). This is in concordance with our results.

Jankowski et al. [15] conducted a retrospective study over a 7-year period (2001–2008) focusing on the incidence of and risk factors associated with LRRC. In their cohort, the local recurrence rate was 7.4% (27/365). Their analysis revealed that the subgroup treated exclusively with short-term radiotherapy, using 5 × 5 Gy, showed effective local control with a local recurrence rate of only 4.2% [15]. This treatment is not used very often nowadays and is predominantly administered in older patients who cannot receive chemotherapy. At present, neoadjuvant radio-chemotherapy or total neoadjuvant therapy are the most common preoperative therapy regimes for locally advanced rectal cancer [16].

However, there are multiple potential risk factors for local recurrence after rectal cancer resection besides the absence of neoadjuvant treatment. Jankowski et al. identified a low location in the rectum of the primary tumor as another relevant risk factor for local recurrence [15]. In our study cohort, over one third of the patients with LRRC, (14/36; 38.9%) had their primary tumor located in the lower third of the rectum. The circumferential resection margin (CRM) was involved in one of these 14 patients, whereas ten patients had a negative CRM; furthermore, one patient underwent local excision, and CRM data were missing in two instances. In contrast, only 16.7% (6/36) had their primary tumor in the upper third of the rectum, with three of the six patients having a negative CRM, while CRM status was unknown in the remaining three patients. This risk factor may be due to the narrow anatomy in the pelvis with closer resection margins even when a total mesorectal excision (TME) is correctly performed in the lower third of the rectum.

Another retrospective single center study from Mayo Medical Center, Rochester, Minnesota, conducted by Hahnloser et al. found no correlation between initial tumor treatment and local recurrence. This investigation included a cohort of 429 patients diagnosed with LRRC between 1981 and 1996 [17]. All subjects had previously undergone curative surgery and received multimodal treatment for their recurrent tumor. After restaging, 304 patients were thought to be eligible for surgery, yet only 138 patients underwent curative resection (R0). A total of 139 patients had palliative resection due to gross tumors that could not be entirely excised due to technical limitations and 27 patients showed microscopically residual tumor mass. Looking at these subgroups, survival was best when complete tumor excision was achieved (5-year survival 37%), second best in the presence of only microscopically residual tumor mass (5-year survival 22%) and poorest when larger masses (R2) of tumor remained (5-year survival 16%). Although these findings are derived from an earlier period and advancements in radio-chemotherapy as well as surgical techniques have occurred since then, it remains evident that achieving R0 resection is the most important principle in successful treatment of LRRC.

This conclusion is supported by Caricato et al. in their meta-analysis regarding prognostic factors after surgery for LRRC [18]. They identified eight studies confirming that R0 resection was a statistically significant prognostic factor yielding better survival outcomes compared to R1 and R2 resections. This is further supported by a national Swedish analysis conducted by Westberg [19]. They included all patients with LRRC in the period between 1995 and 2002 using data from their nationwide colorectal cancer registry. They reported a 5-year survival rate of 43% for patients after R0 resection and 14% for patients after R1 resection.

A systematic review by Fadel et al. [20] regarding oncological outcomes following multimodal treatments and surgery in patients with LRRC determined that a neoadjuvant treatment for recurrence is highly beneficial and should be implemented as a combination of radiation and chemotherapy whenever possible. Furthermore, Fadel et al. [20] reported that neoadjuvant radio-chemotherapy was helpful in achieving an R0-resection and that patients even benefited from neoadjuvant radiation alone (followed by radical resection) compared to upfront surgery with potential adjuvant systemic treatment. Likewise, Rödel et al. found that 8% of patients with recurrent cancer, who were initially deemed nonresectable could be subjected to surgery after neoadjuvant radio-chemotherapy [21].

As Klose et al. showed in their multivariate analysis of 90 patients with LRRC, curative resection is more likely for intraluminal local recurrence compared to extraluminal recurrence and it is significantly associated with prolonged survival [22]. The fact that almost half of our cohort (15 patients, 41.7%) had a recurrence at the previous anastomotic site may at least partially elucidate the good R0 resection rate of 54.5% (12/22), which is reflected in the good survival outcomes. Rahbari and colleagues have also shown that R0 resection of LRRC is a decisive prognostic determinant for survival, even in the presence of extrapelvic tumor recurrence: in their multivariate analysis of 107 patients with LRRC, R0 resection could be achieved in 58.7%. The R0 rate is thus comparable to the rate of 54.5% described by us. Three- and 5-year survival rates were 61% and 47%, which are lower relative to our cohort. In their multivariate analysis, surgical morbidity, presence of extrapelvic disease, and R1/2 resection were independent predictors of poorer survival [23].

We observed a 3-year overall survival of 79% without separating the patients into groups due to the small sample size. Our results align with those of Kim et al. [11] who conducted a retrospective analysis of patients with early and late recurrent rectal cancer in Asia. They reported a 3-year overall survival of 76% for the early and 90% for the late recurrence group.

Another study by Agas et al. [24] described a relatively poor overall survival rate for locally recurrent rectal cancer patients (68%, 41%, 37% for 3-, 5-, 7-year overall survival (OS), respectively), who were eligible for surgery and had undergone intraoperative radiotherapy (IORT).

Furthermore, Fadel et al. [20] revealed a reduced 5-year OS in their meta-analysis compared to our and Kim’s results. They included 14 retrospective studies and one prospective study on treatment and survival in locally recurrent rectal cancer patients. The patient cohort could be divided into 4 different treatment groups and reported a 5-year OS of: 35% for the neoadjuvant radio-chemotherapy group, 30% for the surgery only group, and 29% for the neoadjuvant radiotherapy group. The poorest 5-year survival was seen in the adjuvant chemo-radiotherapy group with 21%. This is not in line with our results, which might be due to older data (the included studies were published between 1997 and 2006) and other treatments and chemotherapeutics provided in their study cohort. Our results showed that the recurrent tumor mass predominantly recurred at the former tumor site or anastomotic site, which is also described by Park et al. [25]. They report similar recurrence patterns as found in our study cohort. The central type of pelvic recurrence was the most common (*n* = 21, 33.9%) followed by lateral (*n* = 14, 22.6%), posterior (*n* = 13, 21%), perineal (*n* = 8, 12.9%), and anterior types (*n* = 6, 9.7%). This is in accordance with our results which showed a central recurrences rate of 41% (15/36), lateral recurrence rate of 25% (9/36), and posterior recurrence rate of 22% (8/36). The results differ only in regard of LRRC affecting the anterior region, where we observed a considerably elevated recurrence rate of 30% (11/36) compared to 9.7%.

A large systematic review suggests that the risk of local recurrence of colorectal cancer triples after postoperative anastomotic leakage [26]. This is not fully reflected in our data: only one of the 36 patients with LRRC had anastomotic leakage after primary surgery. Another independent risk factor for local recurrence is CRM involvement [27]. Again, our data are not entirely conclusive, partly because no data on CRM were available from the external primary surgeries: CRM was negative in 21 of 36 patients (58.3%), in 13 (36.1%) there were no CRM data available, and a local procedure was conducted in two patients. However, the R status was not specified in two cases, whereas in 32 of 36 patients (88.9%), an R0 resection was accomplished, and in two patients, R1 was reported. There were no cases of R2 resection. It can therefore be concluded that most local relapses in our cohort occurred despite previous R0 resection and negative CRM.

Our study has several limitations. Firstly, selection bias cannot be excluded due to the retrospective study design. Secondly, despite the long observation period, the sample size is small. Nevertheless, the strengths of our study are also evident: the data summarize a period of almost 12 years of treatment of rectal cancer recurrences at a nationally recognized colorectal surgery center. This can contribute to filling the evidence gaps regarding therapeutic algorithms for LRRC.

## Conclusion

LRRC remains a challenging topic, even in interdisciplinary teams and for highly educated and trained experts. With an individualized therapeutic approach, an R0 resection of LRRC can be achieved in almost two-thirds of cases. The resulting good 5-year survival rate of 72% supports a multimodal therapeutic approach with surgical resection of the local recurrence, with acceptable postoperative morbidity. To tailor multimodal therapy even more precisely and specifically to the individual localization and tumor biology, further findings from prospective studies with large numbers of cases are required.

## Data Availability

The data that support the findings of this study are available on request from the corresponding author.
